# Identification of functional TFAP2A and SP1 binding sites in new TFAP2A-modulated genes

**DOI:** 10.1186/1471-2164-11-355

**Published:** 2010-06-03

**Authors:** Francesca Orso, Davide Corà, Benedetta Ubezio, Paolo Provero, Michele Caselle, Daniela Taverna

**Affiliations:** 1 Molecular Biotechnology Center (MBC) and Department of Oncological Sciences, University of Torino, Via Nizza, 52, 10126 Torino, Italy; 2 National Institute of Nuclear Physics (INFN) and Department of Theoretical Physics, University of Torino, Via Pietro Giuria, 1, 10125, Torino, Italy; 3 Molecular Biotechnology Center (MBC) and Department of Genetics, Biology and Biochemistry, University of Torino, Via Nizza, 52, 10126 Torino, Italy; 4 Center for Complex Systems in Molecular Biology and Medicine, University of Torino, Via Acc. Albertina, 13, 10023 Torino; Italy; 5 Systems Biology Lab, Institute for Cancer Research and Treatment (IRCC), School of Medicine, University of Torino, Str. Prov. 142 Km. 3.95, I-10060 Candiolo, Torino, Italy; 6 Cancer Research UK London Research Institute, Lincoln's Inn Fields Laboratories, Vascular Biology Laboratory, 44 Lincoln's Inn Fields, London, WC2A 3PX, UK

## Abstract

**Background:**

Different approaches have been developed to dissect the interplay between transcription factors (TFs) and their cis-acting sequences on DNA in order to identify TF target genes. Here we used a combination of computational and experimental approaches to identify novel direct targets of TFAP2A, a key TF for a variety of physiological and pathological cellular processes. Gene expression profiles of HeLa cells either silenced for TFAP2A by RNA interference or not were previously compared and a set of differentially expressed genes was revealed.

**Results:**

The regulatory regions of 494 TFAP2A-modulated genes were analyzed for the presence of TFAP2A binding sites, employing the canonical TFAP2A Positional Weight Matrix (PWM) reported in Jaspar http://jaspar.genereg.net/. 264 genes containing at least 2 high score TFAP2A binding sites were identified, showing a central role in "Cellular Movement" and "Cellular Development". In an attempt to identify TFs that could cooperate with TFAP2A, a statistically significant enrichment for SP1 binding sites was found for TFAP2A-activated but not repressed genes. The direct binding of TFAP2A or SP1 to a random subset of TFAP2A-modulated genes was demonstrated by Chromatin ImmunoPrecipitation (ChIP) assay and the TFAP2A-driven regulation of DCBLD2/ESDN/CLCP1 gene studied in details.

**Conclusions:**

We proved that our computational approaches applied to microarray selected genes are valid tools to identify functional TF binding sites in gene regulatory regions as confirmed by experimental validations. In addition, we demonstrated a fine-tuned regulation of DCBLD2/ESDN transcription by TFAP2A.

## Background

The coordination of various complex biological functions as well as the response to environmental and developmental stimuli are governed by biochemical processes that regulate gene activity. Transcription is the initial step of gene expression and it involves a multitude of transcription factors (TFs), their corresponding *cis*-acting elements on DNA, additional co-factors and the influence of chromatin structure [1]. Functional TF binding sites (TFBSs) can be identified in the genome by computational approaches or experimentally by Chromatin ImmunoPrecipitation and hybridization on a genomic microarray (ChIP on Chip) [2] or by high-throughput selection procedures (SELEX) in which pools of random DNA sequences are mixed with a TF and those that are preferentially bound are recovered and sequenced [3,4]. However, an alternative and very promising approach consists in combining *in silico *TFBS predictions in the gene promoter regions and microarray analyses, comparing gene expression of cells in which a TF is either over-expressed or deleted [5-7]. Indeed, the analysis of regulatory sequences of putative co-regulated genes might be useful in identifying common *cis*-regulatory elements recognized by specific TFs [5]. The microarray assays help to narrow down the number of genes to be analyzed, focusing on those more likely to be regulated by the same TFs, thus reducing the false positive and negative rates.

The Activator Protein-2 (TFAP2) family of transcription factors includes five different yet closely related proteins known as TFAP2A, TFAP2B, TFAP2C, TFAP2D and TFAP2E [8-12] encoded by different genes. TFAP2 can positively or negatively regulate the promoter activity of many pivotal genes involved in physiological or pathological processes such as development, cell growth, differentiation, apoptosis and tumorigenesis [12]. Among the positively regulated genes are: *CDKN1A*, *TGFA*, estrogen receptor, keratinocyte-specific genes, *KIT*, *HIV KTF1*, *HTLVI*, type IV collagenase, SV40 enhancer region, human metallothionein gene IIa, *ERBB2*, *IGFB5*, dopamine beta-hydroxylase. Examples of repressed genes are: *MCAM, CEBPA *and *MYC *[12]. The crucial role of the TFAP2 genes in regulating fundamental biological processes is highlighted by the embryonic lethality of the genetically modified Tcfap2a or Tcfap2b or Tcfap2 g mice [12,13].

Every TFAP2 protein possesses a unique, highly conserved helix-span-helix dimerization motif at the C-terminal half of the protein, a central basic region and a less conserved proline- and glutamine-rich domain at the amino terminus [14]. The helix-span-helix motif and the basic region mediate DNA binding and dimerization [15] while the proline- and glutamine rich region is responsible for transcriptional transactivation. The TFAP2 proteins are able to form hetero- as well as homo-dimers and bind to GC-rich DNA sequences within regulatory regions of their target genes, mediating both activation and repression of gene transcription [12]. Functional TFAP2 binding sites, such as 5'-GCCN3GGC-3' or 5'-GCCN4GGC-3' or 5'-GCCN3/4GGG-3' have been identified [16]. However other well characterized binding sites, such as 5'-CCCCAGGC-3' [17] or others [18], which differ considerably from the previous sequences, have also been found, indicating that TFAP2 binding sites may represent promiscuous GC-rich elements varying considerably in binding affinity. This makes the computational identification of TFAP2 binding sites not a trivial process. A Positional Frequency Matrix (PFM) obtained by multiple alignment algorithms, which leads to nucleotide scores indexed by letters and positions is often used to localize degenerated *cis*-regulatory elements [19]. In addition, given that TFAP2 isoforms are very similar in their DNA binding domains, a specific sequence preference between different TFAP2 proteins has not been found, as demonstrated by an in vitro binding site selection with recombinant TFAP2A and TFAP2C proteins [20].

Several molecular mechanisms control the TFAP2 protein activity and physical interactions with other proteins play an important role. Among the most important known TFAP2 interacting proteins, we can list DNA binding factors such as YY1 [21]; YB1 [22]; TP53 [23]; SP1 [24]; MYC [25]; PAX6 [26]; RB1 [25]; CUX1 [27]; viral proteins such as SV40 large T antigen, 1 human T-cell leukemia virus type 1 [28] and adenovirus E1A protein [29] as well as non-DNA-binding factors such as WWOX [30]; GAS41 [31]; PARP1 [32]; APC [33]; CREB [34]; CITED2 and 4 [35]; PC4 [36]; DEK [37] and YAP [38].

We previously performed whole-genome microarray analysis for HeLa cells either silenced for TFAP2A by RNA interference or not and identified a set of differentially expressed genes [39]. The regulatory regions (-900/+100, considering the TSS as +1) of the genes that unambiguously mapped to known ENSEMBL IDs were analyzed for the presence of TFAP2A binding sites, employing the canonical Positional Weight Matrix (PWM) reported in Jaspar http://jaspar.genereg.net/. 264 genes containing at least 2 high score TFAP2A binding sites were identified, several of which could be validated by Chromatin ImmunoPrecipitation (ChIP) assays. Additionally, a detailed analysis of the TFAP2A-driven regulation of the Discoidin, CUB and LCCL domain containing 2/Endothelial and Smooth muscle Derived Neuropilin like/CUB, LCCL-homology, coagulation factor V/VIII homology domains (*DCBLD2/ESDN/CLCP1*) gene was performed. Finally we searched for TFs that might cooperate with TFAP2A in the transcriptional regulation of genes containing at least 2 high score TFAP2A binding sites and found SP1 as a potential candidate for TFAP2A activated genes.

## Results

### Identification of TFAP2A binding sites in newly identified TFAP2A-modulated genes

**Figure 1:**
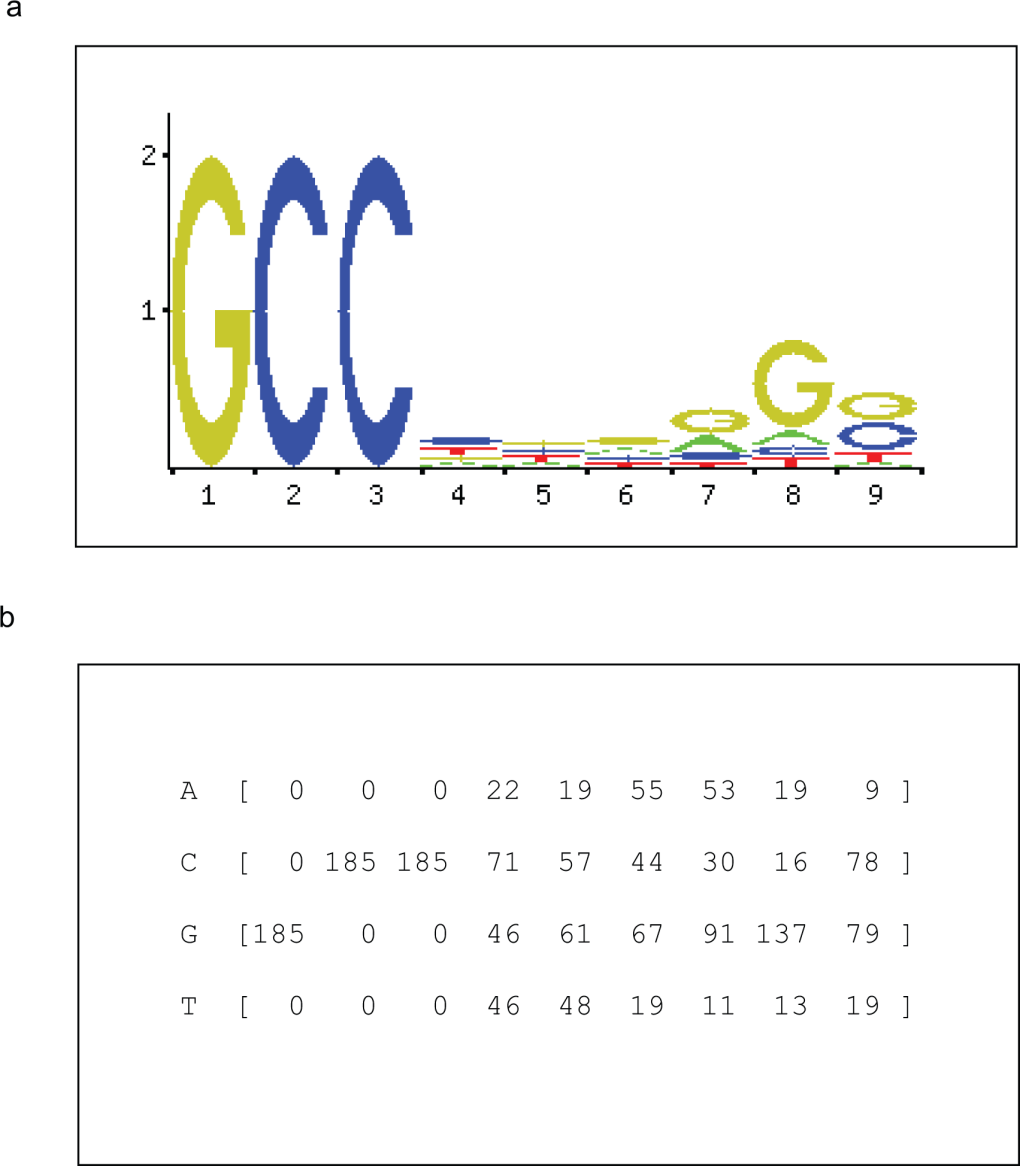
**Sequence logo and canonical Positional Weight Matrix (PWM) of TFAP2A**. Logo (A) and frequencies (B) defining the TFAP2A PWM MA0003 are shown as provided by Jaspar database http://jaspar.genereg.net/.

In order to define TFAP2A binding sites in newly identified TFAP2A-modulated genes [39] we first assembled a dataset of core promoter regions (-900/+100, considering the TSS as +1) for all known human protein-coding genes (21316) using the ENSEMBL database and searched for TFAP2A binding sites employing the canonical TFAP2A Positional Weight Matrix (PWM) MA0003 (Figure 1) reported in the Jaspar database. Affinity scores were assigned using standard log-likelihood ratios [40] and a binding site defined as an oligonucleotide with log-likelihood ratio higher than 66% of the maximum score possibly associated to the PWM. After ranking the binding sites by score, we used various thresholds (top-scoring 10%, 20% and 30% sites) to classify the genes containing at least one or two high score TFAP2A binding sites (Table 1). In the following we will mostly consider the top-scoring 20% binding sites. We then focused on the set of the differentially expressed genes identified by microarray analysis [39], in which gene expression profiles of HeLa cells, either silenced for TFAP2A by RNA interference or not were compared considering a Fold Change (FC) > ± 1.5 and a p value (p_v_) < 0.01. For each of them the longest available transcript was chosen (see Additional file 1). Significant enrichment for TFAP2A binding sites was found in the regulatory regions of these genes compared with genome-wide abundance as calculated using an exact Fisher test, as shown in Table 1. In the whole genome the genes containing at least one or two high score TFAP2A binding sites were respectively 12686 and 8636 whereas, among TFAP2A-regulated genes, 363 out of 494 genes (ENSEMBL) contained at least one high score TFAP2A binding site while 264 out of 494 (157 down- and 107 up-regulated) genes (ENSEMBL) contained a minimum of two sites indicating an enrichment for TFAP2A binding sites in the TFAP2A-regulated genes (p_v _= 1.5E-05), see Table 1. The results for different thresholds (top 10% and 30%) were similarly significant and shown in Table 1. We ranked the genes according to the number of TFAP2A binding sites present in their core promoter regions (Table 2) and found that the majority of the genes contained one or two TFAP2A binding site/s. It's important to underline that already reported TFAP2A target genes were identified in our analysis (see Additional file 1).

**Table 1:** Identification of TFAP2A binding sites in the core promoter of candidate TFAP2A-modulated genes.

Minimum number of binding sites per gene	TFA2A binding site score threshold	Genome-wide	TFAP2A-regulated genes	p (Fisher test)
1	All sites	19402	468	
	
	best 30% (≥11.02)	14710	401	7.03E-08
	
	best 20% (≥11.44)	12686	363	4.56E-09
	
	best 10% (≥12.14)	9416	283	1.02E-07

2	All sites	17540	447	
	
	best 30% (≥11.02)	10959	322	1.03E-05
	
	best 20% (≥11.44)	8636	264	1.53E-05
	
	best 10% (≥12.14)	4955	155	2.0E-03

Number of genome-wide or candidate TFAP2A-modulated human protein-coding genes containing at least one or two high score TFAP2A binding sites defined at various score thresholds (10%, 20%, 30%) in their core promoters. By considering only high-scoring sites we obtained a significant enrichment in TFAP2A-modulated genes compared with total binding sites, as shown by the p-values in the last column (exact Fisher Test).

**Table 2:** Distribution of genes containing TFAP2A binding sites.

	Genes			
	**Genome-wide**		**TFAP2A-regulated**	

**Minimum number of binding sites per gene best 20% (≥11.44)**	**n°**	**%**	**n°**	**%**

1	12686	65.36	363	77.56

2	8636	44.50	264	56.41

3	5967	30.75	185	39.53

4	4131	21.30	133	28.42

5	2816	14.51	102	21.79

6	1948	10.04	72	15.38

7	1339	6.90	51	10.90

8	920	4.74	35	7.48

9	646	3.33	22	4.70

10	448	2.31	14	2.99

11	297	1.53	12	2.56

12	195	1.00	7	1.50

13	129	0.66	4	0.85

14	85	0.44	1	0.21

15	57	0.29	0	0

16	31	0.16	0	0

17	20	0.10	0	0

18	14	0.07	0	0

19	8	0.04	0	0

21	5	0.03	0	0

22	3	0.01	0	0

26	2	0.01	0	0

27	1	0.01	0	0

Distribution of genome-wide or TFAP2A-modulated genes based on absolute number of genes (or percentages, %) containing one or more high score (best 20%) TFAP2A binding sites in their core promoters.

### Functional classes enrichment for predicted TFAP2A target genes

**Figure 2:**
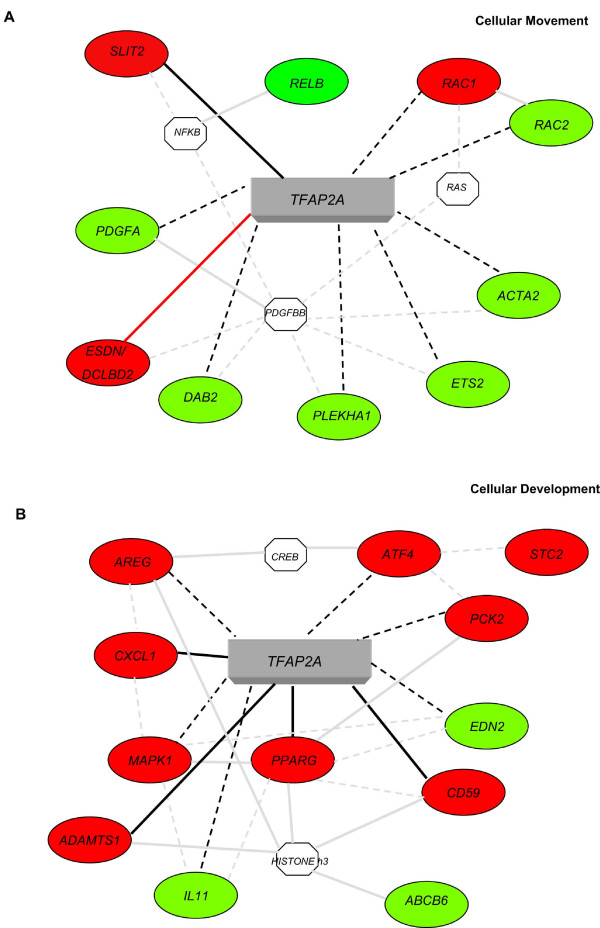
**Ingenuity Pathway Analysis Systems for the genes containing at least 2 high score TFAP2A binding sites**. Specific functional networks for the 264 genes containing at least 2 high score TFAP2A binding sites were obtained using Ingenuity Pathway Analysis Systems. A simplified representation of the two main molecular networks identified, "Cellular Movement" (A) and "Cellular Development" (B) is shown. Legend: Up- or down-regulated genes are shown in red or green respectively; continuous grey lines indicate direct interactions experimentally proven in literature; dashed grey lines represent potential indirect connections; continuous black lines represent direct connections demonstrated by our microarray and ChIP analyses; dashed black lines represent potential direct connections demonstrated by our microarray results [39]. Octagons indicate genes absent in the 264 gene dataset but related to it as indicated from the literature. Red lines indicate direct connections demonstrated by our microarray, ChIP and promoter analyses.

To identify the functional pathways in which the potential TFAP2A targets could be involved Gene Ontology (GO) and network analyses were performed for the 264 TFAP2A-modulated genes containing at least two TFAP2A high score binding sites using the Ingenuity Pathway Analysis Systems. Two high score molecular networks were identified and in Figure 2A and 2B we show a selection of these genes and their connections with TFAP2A. The first network associated with Cellular Movement (Figure 2A, score 38) and included 26 genes, i.e. *SLIT2 *(slit homolog 2 -Drosophila); *PDGFA *(platelet-derived growth factor alpha polypeptide); *RAC1 *and *RAC2 *(ras-related C3 botulinum toxin substrate 1 and 2, rho family, small GTP binding protein Rac1 and Rac2); *DCBLD2/ESDN *(discoidin, CUB and LCCL domain containing 2/Endothelial and Smooth muscle cell Derived Neuropin-like molecule); *ACTA2 *(actin, alpha 2, smooth muscle, aorta). The second network associated with Cellular Development (Figure 2B, score 38) and included 24 genes, i.e. *PPARG *(peroxisome proliferator-activated receptor gamma); *MAPK1 *(mitogen-activated protein kinase 1); *CXCL1 *(chemokine, C-X-C motif, ligand 1 - melanoma growth stimulating activity, alpha); *ADAMTS1 *(metallopeptidase with thrombospondin type 1 motif, 1); *AREG *(amphiregulin); *IL11 *(interleukin 11).

### Functional validation of potential TFAP2A-regulated genes

**Figure 3:**
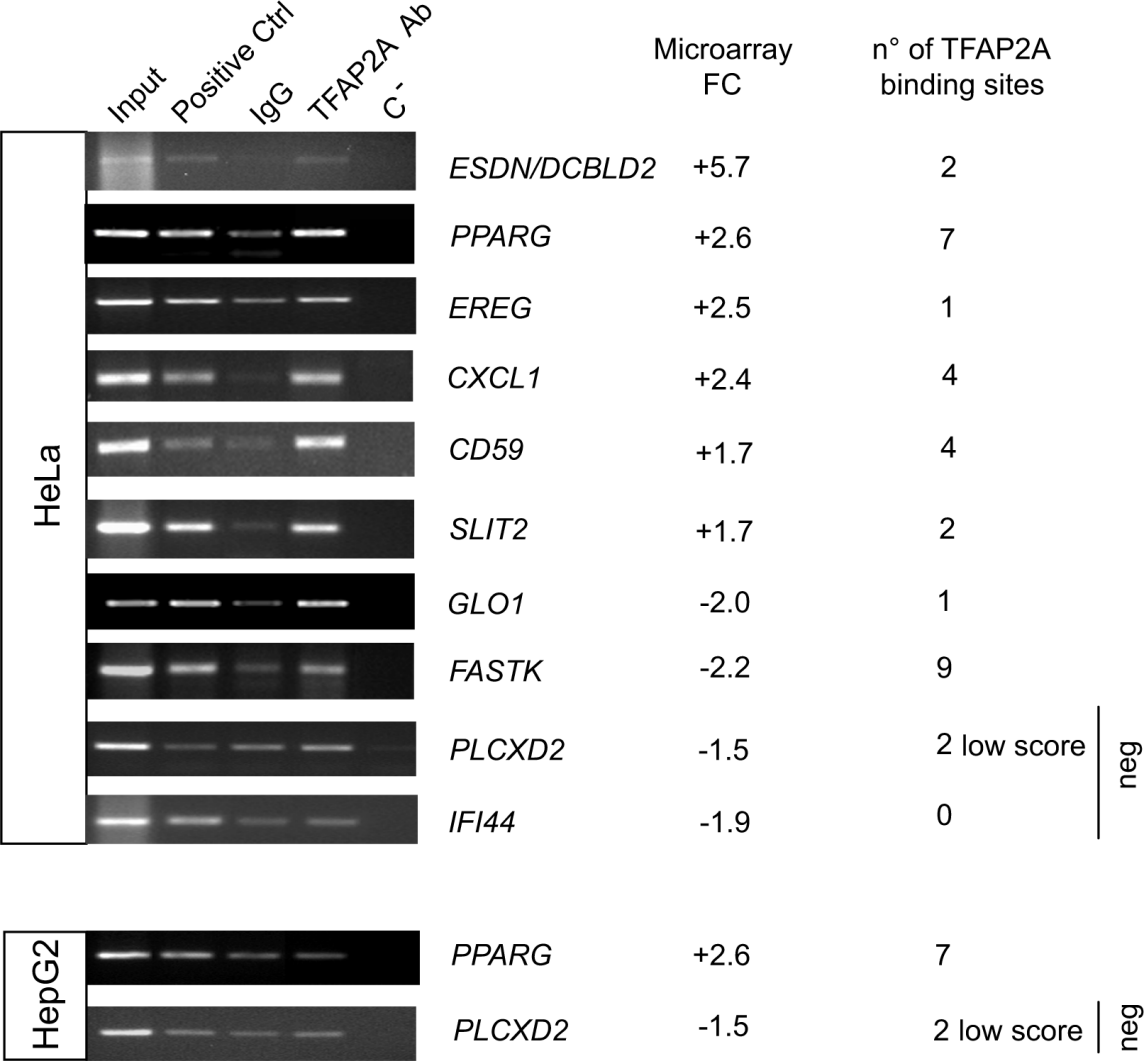
**In vivo Chomatin ImmunoPrecipitation (ChIP) analysis for putative TFAP2A binding sites on the core promoters of potential TFAP2A target genes**. Pre-cleared chromatin from HeLa or HepG2 cells was immunoprecipitated with either non-specific IgG (IgG, negative control) or anti-RNA polymerase II or anti-acetylhistone H3 (positive controls: Positive Ctrl) or anti-TFAP2A (TFAP2A) antibodies. Immunoprecipitated DNA or non-immunoprecipitated chromatin samples (input) were amplified by PCR using primer pairs designed with the NCBI Primer Designing Tool (see Methods) across the TFAP2A putative binding sites. ***CD59***: CD59 molecule, complement regulatory protein; ***CXCL1***: chemokine (C-X-C motif) ligand 1 (melanoma growth stimulating activity alpha); ***DCLBD2/ESDN***: endothelial and smooth muscle cell derived neuropilin like molecule; ***EREG***: epiregulin; ***FASTK***: fast kinase; ***GLO1***: glyoxalase I; ***IFI44***: interferon-induced protein 44; ***PLCXD2***: pleckstrin homology-like domain family B member 2; ***PPARG***: peroxisome proliferator activated receptor gamma; ***SLIT2***: slit homolog 2 (Drosophila). C-, PCR negative control; FC, fold changes obtained from the microarray analysis [39]. Number (n°) of TFAP2A binding sites identified computationally. Three independent experiments were performed and a representative one is shown here.

Potential TFAP2A binding was tested for 13 candidate target genes containing at least one or two best 20% TFAP2A binding sites by Chromatin Immuno Precipitation (ChIP) assay (Figures 3 and 6): *ADAMTS1 *(ADAM metallopeptidase with thrombospondin type 1 motif, 1); *CASP9 *(caspase 9); *CD59 *(CD59 molecule, complement regulatory protein); *CXCL1 *(chemokine ligand 1, melanoma growth stimulating activity alpha); *EREG *(Epiregulin); *DCBLD2/ESDN *(endothelial and smooth muscle cell derived neuropiline like molecule); *FASTK *(Fas-activated serine/threonine kinase); *GLO1 *(glyoxalase I); *KRT16 *(keratin 16); *KRT17 *(keratin 17); *PPARG *(peroxisome proliferator activated receptor gamma); *SLIT2 *(slit homolog 2, Drosophila); *TGFBI *(Transforming Growth Factor B-Induced). The ENSEMBL ID, microarray Fold Change (FC) and TFAP2A binding sequences, scores and positions for each of these genes is shown in Table 3. ChIP analysis was performed on HeLa cells, that endogenously express TFAP2A and as shown in Figure 3 and 6, enrichment for TFAP2A was found on the promoter of each gene compared with negative controls, suggesting in vivo binding and direct regulation of these genes by TFAP2A. Negative controls for ChIP analysis were performed using genes in which low score or no TFAP2A binding sites were identified such as *PLCXD2 *(pleckstrin homology-like domain family B member 2) or *IFI44 *(interferon-induced protein 44). In fact, no enrichment for TFAP2A was observed in the promoter of these two genes compared with the negative IgG controls suggesting that genes containing only low score or no TFAP2A binding sites are not direct TFAP2A targets and their TFAP2A-dependent modulation is indirect. ChIP analysis for *PPARG *and *PLCXD2 *genes was also performed in HepG2 cells that do not express TFAP2A and no enrichment for TFAP2A was observed for any of the analyzed sequences supporting the significance of the results obtained in HeLa cells (Figure 3 and 6).

**Table 3:** TFAP2A binding site sequences present in the regulatory regions of some TFAP2A- modulated genes.

Gene Symbol	ENSEMBL ID	Microarray FC	TFAP2A binding sequence	TFAP2A binding score	Position
*ESDN/DCBLD2*	ENSG00000057019	5.7	GCCGCGGGG	12.74	-269

			GCCCGCAGC	11.54	-10

*PPARG*	ENSG00000132170	2.6	GCCTGAGGC	11.48	-851

			GCCGGGGGC	12.82	-280

			GCCGCGGGG	12.74	-176

			GCCCCGCGG	11.77	-175

			GCCGTGGGC	11.94	-134

			GCCCGGCGC	11.85	+10

			GCCCGCGGC	12.85	+42

*EREG*	ENSG00000124882	2.5	CCCTCGGGC	12.75	-101

*CXCL1*	ENSG00000163739	2.4	GCCCGGGGG	13.46	-64

			GCCCCCGGG	12.76	-63

			GCCCGGAGC	12.14	-56

			GCCGCAGGC	11.91	+13

*CD59*	ENSG00000085063	1.7	GCCCTGGGG	12.58	-336

			GCCCCAGGG	12.55	-335

			GCCGGGAGC	11.51	-37

			GCCGGGGGG	12.83	+3

*ADAMTS1*	ENSG00000154734	1.7	GCCCGCAGC	11.54	-142

			GCCGGGGGC	12.82	-107

*SLIT2*	ENSG00000145147	1.7	GCCGGGGGC	12.81	-679

			GCCCCGAGG	12.06	-248

*CASP9*	ENSG00000132906	-1.5	GCCCTGGGG	12.58	-734

			GCCCCCAGG	11.45	-463

			GCCCCCAGG	11.45	-370

			GCCCGCAGG	11.55	-182

			GCCCTGGGG	12.58	+4

			GCCCCCAGG	11.45	+69

			GCCCCGCGC	11.75	+2

*TGFBI*	ENSG00000120708	-1.6	GCCCTGGGG	12.58	-899

			GCCCCCAGC	11.44	-414

			GCCCTGGGC	12.57	-212

			GCCCTGGGC	12.57	+43

*GLO1*	ENSG00000124767	-2.0	GCCGCGGGC	12.72	-24

*FASTK*	ENSG00000164896	-2.2	GCCCGGAGG	12.15	-856

			GCCCCGAGC	12.04	-500

			GCCCGGGGC	13.45	-413

			GCCCCGGGG	13.36	-393

			GCCCCCGGG	12.76	-391

			GCCCTCGGC	11.97	-66

			GCCCGGCGC	11.85	-59

			GCCCGCGGG	12.86	-42

			GCCGGGAGC	11.51	-2

*KRT16*	ENSG00000186832	-2.5	GCCCTCGGG	11.98	-860

			GCCCCCGGG	12.76	-650

			GCCTGAGGC	11.48	-502

			GCCCCGAGG	12.06	-279

			GCCCTCGGG	11.98	-277

*KRT17*	ENSG00000186831	-2.7	GCCCCGGGG	13.36	-525

			GCCCCGGGG	13.36	-524

			GCCCCCAGC	11.44	-203

			GCCTGGGGG	12.30	+56

*PLCXD2*	ENSG00000144824	-1.5	CCCCCTTGGC	9.47*	-878

			GGCCAGGGC	9.04*	-64

*IFI44*	ENSG00000137965	-1.9	-	-	-

For each ChIP-validated TFAP2A target gene or negative control (see figure 3 and 6) Gene Symbol, ENSEMBL IDs, TFAP2A binding site sequence, score and position (referring to the TSS as +1) are shown. ***ADAMTS1***: ADAM metallopeptidase with thrombospondin type 1 motif, 1; ***CASP9***: caspase 9; ***CD59***: CD59 molecule, complement regulatory protein; ***CXCL1***: chemokine (C-X-C motif) ligand 1 (melanoma growth stimulating activity alpha); ***DCLBD2/ESDN***: Endothelial and smooth muscle cell derived neuropiline like molecule; ***EREG***: Epiregulin; ***FASTK***: fast kinase; ***GLO1***: glyoxalase I; ***KRT16***: keratin 16; ***KRT17***: keratin 17; ***IFI44***: interferon-induced protein 44; ***PLCXD2***: pleckstrin homology-like domain family B member 2; ***PPARG***: peroxisome proliferator activated receptor gamma; ***SLIT2***: slit homolog 2 (Drosophila); ***TGFBI***: Transforming Growth Factor Beta-Induced. * low score binding sites, used as negative controls.

### The *DCBLD2/ESDN/CLCP1 *promoter region is directly regulated by TFAP2A

**Figure 4:**
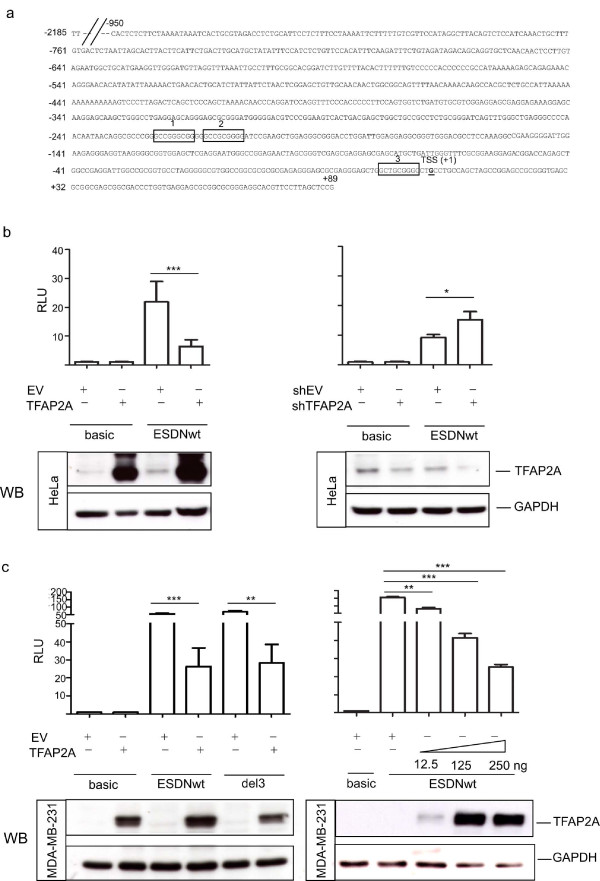
**Regulation of the *DCBLD2/ESDN *promoter by TFAP2A**. (A) The genomic organization of the human *DCBLD2/ESDN *promoter (-2185/+89) is shown and the three highest score TFAP2A binding sites identified are framed and named 1 or 2 or 3. The Transcription Start Site (TSS) is considered +1. (B)(C) HeLa (B) and MDA-MB-231 (C) cells were transiently co-transfected with either pGL3-Basic (basic) or pGL3-ESDN-WT (ESDNWT) or pGL3-ESDN-del3 (del3) vectors together with pSP(RSV)TFAP2A (TFAP2A) or TFAP2AshRNA2 (shTFAP2A) or their respective control empty vectors (EV or shEV) to obtain TFAP2A over-expression or down-modulation. pRLTK (Renilla luciferase) vector was transfected along to evaluate transfection efficiency and perform normalizations. Forty-eight hours later Firefly Luciferase activity was measured and normalized against Renilla Luciferase. Fold Changes were calculated referring to the pGL3-basic control vector and expressed as Relative Luciferase Units (RLUs). 250 ng of TFAP2A or shTFAP2A or EV or shEV were transfected unless specified differently. Three independent experiments were performed in triplicate and a representative one is shown here. The error bars indicate the Standard Errors (SE) of the triplicates. A student's *t *test was performed to evaluate if the experiments were statistically significant. * p_v _< 0.05; ** p_v _< 0.01; *** p_v _< 0.001. TFAP2A levels were measured by Western Blot (WB) analysis. Glyceraldheyde-3-phosphate-dehydrogenase (GAPDH) expression was used for protein loading controls.

*DCBLD2/ESDN/CLCP1 *(discoidin, CUB and LCCL domain containing 2/Endothelial and Smooth muscle cell Derived Neuropin-like molecule/CUB, LCCL-homology, coagulation factor V/VIII homology domains protein) turned out to be the most highly modulated (repressed) gene in our microarray analysis on HeLa cells (FC + 5.7) and to have an important role in cell migration [39]. For these reasons we carried on a detailed computational analysis of the *DCBLD2/ESDN *regulatory region, extended to (-2185/+89 with respect to the TSS), and found high enrichment of GC content and no TATA box around the TSS, features which are common to the core promoters of TFAP2A targets identified with our computational analysis. This sequence was analyzed to position potential TFAP2A binding sites using the canonical TFAP2A Positional Weight Matrix (PWM) as in Jaspar database (see above). Three highly scored TFAP2A binding sites were identified in the region -360/+89. A schematic representation of the 2.185 Kb *DCBLD2/ESDN *promoter is shown in Figure 4A. This region was amplified from a BAC genomic clone (see Methods) and cloned in a luciferase reporter vector generating the pGL3-ESDN-WT (ESDNwt) construct. The effects of TFAP2A on promoter activity were tested by performing reporter assays in HeLa and MDA-MB-231 cell lines expressing, respectively, medium and low levels of TFAP2A as assessed by Western Blot (WB) analyses shown in Figure 4B and 4C. Both cell lines were transiently co-transfected with either ESDNwt or its 5' deletant pGL3-ESDN-DEL3 (del3) starting at -950 or pGL3-Basic (basic) control reporter vector and an expression plasmid for TFAP2A, pSP(RSV)TFAP2A (TFAP2A) or its control empty vector (EV) (Figure 4B and 4C). Alternatively HeLa cells (Figure 4B) were transfected with an expression vector for TFAP2A silencing, pSUPER-TFAP2AshRNA2 (shTFAP2A), or with the empty pSUPER control vector (shEV). In addition, cells were transfected with the pRLTK vector for Renilla luciferase expression, to perform transfection efficiency normalization. TFAP2A basal levels, overexpression or silencing were verified by Western Blot (WB) analyses (Figure 4B and 4C) where Glyceraldheyde-3-phosphate dehydrogenase (GAPDH) was used as loading control. 3 fold higher activity was observed for the ESDNwt reporter vector in MDA-MB-231 cells compared with HeLa cells (compare Figure 4B with 4C). The inhibitory function of TFAP2A on *DCBLD2/ESDN *gene transcription was further supported when HeLa and MDA-MB-231 cells were co-transfected with ESDNwt and TFAP2A (250 ng, otherwise specified) with respectively 2 and 3.5 fold reduction in luciferase activity (Figure 4B and 4C). This reduction was inversely proportional to the TFAP2A levels in cells (12.5 or 125 or 250 ng), as shown in Figure 4C for MDA-MB-231 cells. Instead, TFAP2A silencing in HeLa cells caused a 1.6 fold increase in reporter activity (Figure 4B). All together these results strongly suggest a direct repressive activity of TFAP2A on *DCBLD2/ESDN *promoter and are in agreement with our previous microarray results [39].

### Specific role of the TFAP2A binding sites present in *DCBLD2/ESDN *promoter

**Figure 5:**
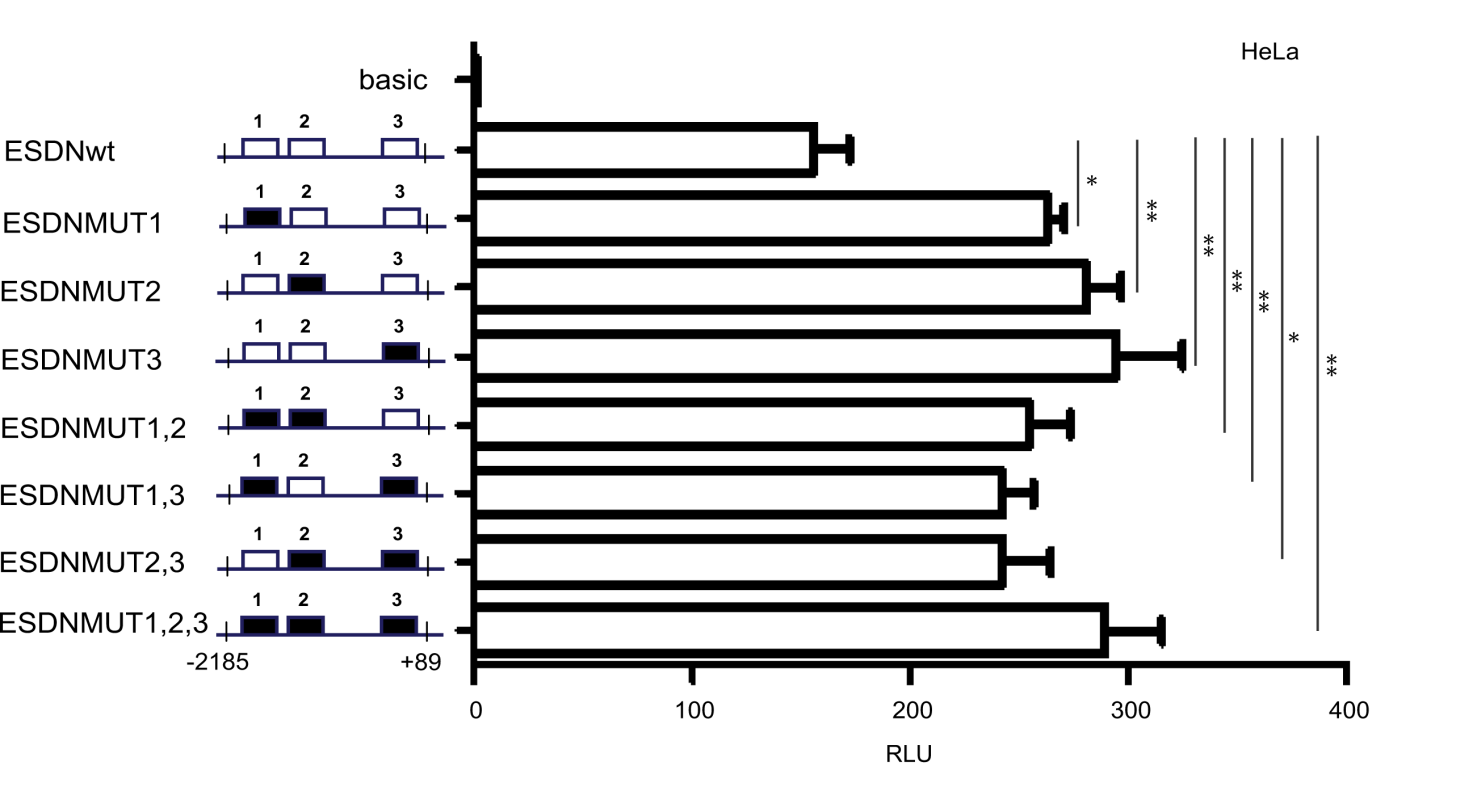
**Functional analysis of the TFAP2A binding sites present in the *DCBLD2/ESDN *promoter**. HeLa cells were transiently transfected with 700 ng of either pGL3-Basic (basic) or pGL3-ESDN-WT (ESDN-WT) or pGL3-ESDN-MUT1 (ESDN-MUT1), pGL3-ESDN-MUT2 (ESDN-MUT2), pGL3-ESDN-MUT3 (ESDN-MUT3), pGL3-ESDN-MUT1,2 (ESDN-MUT1,2), pGL3-ESDN-MUT1,3 (ESDN-MUT1,3), pGL3-ESDN-MUT2,3 (ESDN-MUT2,3), pGL3-ESDN-MUT1,2,3 (ESDN-MUT1,2,3) vectors. pRLTK (Renilla luciferase) vector was transfected as well to evaluate transfection efficiency and perform normalizations. Fold changes were calculated relative to the pGL3-Basic control vector and expressed as Relative Luciferase Units (RLU). Three independent experiments were performed in triplicate and a representative one is shown here. The error bars indicate the Standard Error (SE) of the replicates. * p_v _< 0.05; ** p_v _< 0.01. Wild type binding sites = white boxes; Mutated binding sites = black boxes.

A detailed functional analysis was performed for the main TFAP2A binding site present in *DCBLD2/ESDN *promoter by carrying out site-directed mutagenesis to obtain 7 bp deletions in the central portion of each TFAP2A binding site in single or multiple combinations to generate the constructs reported in Figure 5. Mutations in the TFAP2A binding sites 1 or 2 or 3 (ESDNMUT1 or ESDNMUT2 or ESDNMUT3) produced statistically significant increased promoter activity just like mutations for multiple TFAP2A binding sites 1,2 or 1,3, or 2,3 or 1,2,3 (ESDNMUT1; ESDNMUT1,2; ESDNMUT1,3; ESDNMUT2,3; ESDNMUT1,2,3) as indicated by the student's *t *tests: * p_v _< 0.05; ** p_v _< 0.01 suggesting that each binding site plays a role in repressing the promoter activity.

### Identification of Transcription Factor Binding Sites (TFBSs) present in the promoter regions of TFAP2A-regulated genes by over-represented DNA oligonucleotides or oPOSSUM or MEME analysis

The properties of the core promoter region sequences of the 264 best 20% TFAP2A-regulated genes were studied by using three different approaches based on different biological assumptions and statistical filters such as: 1) short over-represented oligonucleotides; 2) oPOSSUM; 3) MEME.

#### 1. Over-represented oligonucleotides

We performed a genome-wide characterization of the previously described core promoters of human-protein-coding genes, working on the statistical properties of short (5 to 9 nt) DNA oligonucleotides (oligos) present in these sequences. In particular, we identified sets of genes sharing over-represented oligos in their promoter regions according to a binomial model (see Methods for details). We then characterized the evolutionary properties of these oligos using a "conserved over-representation" approach, an alignment-free methodology applied to human-mouse comparison [41]. The resulting different sets of genes were then compared with the up- and down- TFAP2A-regulated gene datasets, described above and the enrichment for oligos in TFAP2A-regulated genes was assessed in the different sets of genes using an exact Fisher test (p_v _< 0.05) as shown in Table 4. Results were ranked according to the corresponding p_v _and the top 10 over-represented oligos are shown in Table 4. For some of these oligos the over-representation is conserved (indicated in Table 4 with an asterisk, *) suggesting an evolutionarily conserved role for them. When possible, a known TFBS consensus was then associated to each oligo, using the list of TRANSFAC TFBS consensus sequences reported in [42]. Interestingly, as shown in Table 4, some of the most over-represented oligos found for the TFAP2A-down-regulated genes correspond to the SP1 consensus sequence (Fisher test: 7.76E-07; 38 target genes). For the up-regulated genes we found many over-represented oligos but we were not able to link them to any known TF.

**Table 4:** Identification of transcription factor binding sites (TFBSs) present in TFAP2A- modulated genes by over-represented DNA oligonucleotides.

TFAP2A-regulated genes	Over-represented oligo	TF	Target genes	p (Fisher test)
DOWN n = 157	CGGGC*	AP-2alpha, C-Rel, MYB, AMEF-2	22	1.20E-07
	
	GAGCCGGC		27	1.48E-07
	
	CCCGC*	OLF-1, AP-2alpha, Sp-1,TCF-1(P),EGR	38	7.76E-07
	
	CCCGGC*	AP-2alpha, MTF-1	47	1.20E-06
	
	CCCCGAGG*	AP-2alpha	51	4.57E-06
	
	CGGCA*	AP-2alpha	57	7.08E-06
	
	CCCATCCTA		64	1.17E-05
	
	CGGCAGCC		65	1.51E-05
	
	GCCCGC*	AP-2alpha	66	2.45E-05
	
	CGGGTGCTC		70	2.75E-05

UP n = 107	AAGCGCG*		6	8.32E-06
	
	GGGGACTAA		11	1.62E-05
	
	AGACGTCT		16	2.04E-05
	
	AAACAACAG*		21	4.27E-05
	
	ACCCACGCG*		26	5.13E-05
	
	CGGGGTAGA		29	5.89E-05
	
	CCTGTTTCG*		33	6.92E-05
	
	CCGGAG		40	7.08E-05
	
	ACGGGTTCT		42	7.59E-05
	
	CCGGAGGC	AP-2alpha	43	8.13E-05

For each TF its consensus binding site sequence, abbreviated name, number of target genes and statistical enrichment values (Fisher-score) are reported. * conserved over-represented oligo

#### 2. oPOSSUM

oPOSSUM [43] is able to evaluate the over-representation of known TFBS on human/mouse conserved regions. We used this tool to analyze the sets of TFAP2A-regulated genes setting the parameters indicated in Methods. Results are reported in Table 5 for the up- and down-regulated genes separately. A strong enrichment was observed for SP1 (Fisher score: 2.19 10E-05; Z-score: 17.47; 95 target genes) and NHLH1 (Fisher score: 7.09 E-05; Z-score: 14.76; 44 target genes) on down-regulated genes. An enrichment for Pax5 (Fisher score: 3.31 E-03; Z-score: 9.98; 12 target genes) and Cebpa (Fisher score: 6.67 E-03; Z-score: 8.20; 41 target genes) was observed for the up-regulated genes even if the statistical relevance was weaker then what observed for the down-regulated genes.

**Table 5:** Identification of transcription factor binding sites (TFBSs) present in TFAP2A- modulated genes by opossum.

TFAP2A-regulated genes	OPOSSUM matrix (Jaspar CORE)	TF	Target genes	p (Fisher test)	Z-score
DOWN n = 157	MA0079	SP1	95	2.19E-05	17.47
	
	MA0048	NHLH1	44	7.09E-05	14.76
	
	MA0006	Arnt-Ahr	99	3.21E-02	14.25
	
	MA0056	MZF1_1-4	119	4.44E-02	11.27
	
	MA0018	CREB1	45	9.48E-03	10.63

UP n = 107	MA0014	Pax5	12	3.31E-03	9.98
	
	MA0102	Cebpa	41	6.67E-03	8.20
	
	MA0042	FOXI1	35	2.12E-01	8.00
	
	MA0135	Lhx3	32	2.11E-01	7.74
	
	MA0047	Foxa2	35	1.93E-01	7.73

For each TF its Positional Weight Matrix (PWM) numbers, abbreviated name, number of target genes and statistical enrichment values (Z-score, Fisher-score) are reported.

#### 3. MEME

MEME is a software for the *ab-initio *identification of relevant motifs in a given set of sequences in which a motif is a sequence pattern that occurs repeatedly in a group of related DNA sequences [44]. The parameters used in our analysis are indicated in Methods and the results obtained are reported in Table 6: MEME regular expression motifs and the relative E-values for the most interesting motifs are indicated. MEME results are not directly associated to known TFBSs and to investigate whether the resulting motifs could be recognized as known TFBSs the same approach used previously for the oligo analysis was used here [42]. For this identification, perfect match between MEME regular expression and the IUPAC equivalent was required, as shown in Table 6, and association between motifs and known TFs was found in some cases. Enrichment for SP1 was also found with this method, in particular for down-regulated genes (E-value: 3.1E-085; 24 target genes).

**Table 6:** Identification of transcription factor binding sites (TFBSs) present in TFAP2A- modulated genes by MEME analysis.

TFAP2A-regulated genes	MEME regular expression	TF	Target genes	MEME E-value
DOWN n = 157	[GA]CCTGTA[AG]TCCCAGC[TA][CA][CT]T[TC]	PITX2, CRX	26	9.1E-162
	
	AT[CT]CTCC[TC][GA]CCTC[AG]G		34	2.6E-058
	
	T[CG][CG]A[GC][TA]CCAGCCTGG[GC]C[AG]AC		29	4.3E-104
	
	AGG[TC]TGCAGTGAGC[CT]G[AT]GAT	Sp-1, AP-2apha, MAZ, TFII-I	24	3.1E-085
	
	GG[GA]GG[CA]GGGG[CGA][GC]GG[GA][GAC]G[GAC]GG		38	1.7E-056
	
	GTGAGCCAC[CT][GA]CGCCCGGC[CT]		21	2.0E-049

UP n = 107	CTCCC[AG]A[AG][GT][TA]GCTGGGA[TC]TA	MYOD, PITX2, CRX	20	2.1E-126
	
	GT[CT]TC[AG]C[TC][AC]TGT[TC][GA][CG]CCAGG	YY1, AP-2alpha	22	1.5E-082
	
	G[GC]GGCGG[GC]G[GC][CG]GG[GC]GG[CG]GG[GC]	Sp-1, AP-2alpha	35	9.7E-062
	
	[GT]TGTGTGTG[TC]G[TC][GA][TC]GTGTG[TG]	MYC	9	3.2E-016
	
	G[GC][TC]TCAAG[CT]GAT[TC]CTCC[TC]GC	NKX2-5	16	1.5E-049
	
	CAGG[CT]G[TC]G[AC]GCCACC[GA]C[GA]CC	AREB6, AML1	19	7.7E-067
	
	ATCT[CT][GA]GCTCACTGCA[AG]CCT		16	9.8E-048
	
	TGGTCTCGA[TA]CTCCTGACCT	T3R, ER	12	8.0E-032

For each TF its consensus binding site sequence, abbreviated name, number of target genes and statistical enrichment values (E-value) are reported.

### Positioning of the SP1 binding sites in the promoter regions of TFAP2A-down-regulated genes and functional validation

After having observed an enrichment for SP1 binding sites in the 157 TFAP2A-down-regulated genes with the three methods mentioned above, we searched for SP1 sites in the promoter regions of these genes using the same approach employed to recognize TFAP2A binding sites: Jaspar, SP1 Positional Weight Matrix MA0079, cut off on the best 20% score. 57 genes containing at least one SP1 binding sites were identified and are listed in Table 7. In the same table it is also indicated if these SP1 motifs were identified with the over-represented oligo or oPOSSUM or MEME approach or not. SP1 binding sites were found in: 2 common genes identified with the oligo analysis and MEME; 12 genes common to oligo analysis and oPOSSUM; 5 genes common to MEME and oPOSSUM; the only common gene to the triple intersection was *OLFML2A*. It is important to underline that SP1 sites did not overlap with TFAP2A binding motifs (data not shown).

**Table 7:** Summary of TFAP2A-down-modulated genes containing SP1 binding sites in their promoters

Down-modulated genes containing SP-1 binding sites					
ENSEMBL ID	Gene symbol	Microarray FC	Oligo	oPOSSUM	MEME
ENSG00000092871	*RFFL*	-1.5	-	-	-

ENSG00000	*SLCO4*	-1.5	-	-	-

ENSG00000	*C16or*	-1.5	-	+	-

ENSG00000	*CDK6*	-1.5	-	+	-

ENSG00000	*GCN5L*	-1.5	-	+	-

ENSG00000	*ATP8B*	-1.5	-	-	-

ENSG00000	*CASP9*	-1.5	+	-	-

ENSG00000	*SPOCK*	-1.5	+	+	-

ENSG00000	*FBN1*	-1.5	-	+	-

ENSG00000	*SMAD3*	-1.5	+	+	-

ENSG00000	*PHLDA*	-1.5	-	+	-

ENSG00000	*DALRD*	-1.5	+	+	-

ENSG00000	*GPRC5*	-1.6	-	-	+

ENSG00000	*KIAA1*	-1.6	-	-	-

ENSG00000	*COL12*	-1.6	-	+	-

ENSG00000	*TGFBI*	-1.6	-	-	-

ENSG00000	*RDH10*	-1.6	+	+	-

ENSG00000	*MESP1*	-1.6	-	-	-

ENSG00000	*OPLAH*	-1.6	+	+	-

ENSG00000	*ST4S6*	-1.6	-	+	+

ENSG00000	*BCOR*	-1.6	+	+	-

ENSG00000	*RPS27*	-1.6	-	+	+

ENSG00000	*SLC25*	-1.6	+	-	-

ENSG00000	*PDGFA*	-1.6	+	+	-

ENSG00000	*DNMT3*	-1.7	-	+	-

ENSG00000	*EPB41*	-1.7	+	+	-

ENSG00000	*CDKN1*	-1.7	+	+	-

ENSG00000	*TMEM1*	-1.7	-	+	-

ENSG00000	*RNF38*	-1.7	-	-	-

ENSG00000	*SLC27*	-1.7	+	-	-

ENSG00000	*SLC43*	-1.7	+	+	-

ENSG00000	*DPYSL*	-1.8	-	+	-

ENSG00000	*SLC7A*	-1.8	-	-	-

ENSG00000	*PXDN*	-1.8	-	-	-

ENSG00000	*COL5A*	-1.8	+	+	-

ENSG00000	*SECTM*	-1.8	+	-	-

ENSG00000	*GLS*	-1.9	-	+	-

ENSG00000	*ENAH*	-1.9	-	+	-

ENSG00000	*FTH1*	-1.9	-	-	-

ENSG00000	*SYNGR*	-2.0	-	+	+

ENSG00000	*TXNIP*	-2.0	-	+	-

ENSG00000	*LOXL2*	-2.0	-	-	-

ENSG00000	*STX6*	-2.0	-	+	+

ENSG00000	*RAB15*	-2.0	+	-	-

ENSG00000	*COX6B*	-2.0	-	-	-

ENSG00000	*OLFML*	-2.0	+	+	+

ENSG00000	*PAG1*	-2.1	-	+	-

ENSG00000	*SESN1*	-2.1	-	+	-

ENSG00000	*ACTA2*	-2.1	-	-	-

ENSG00000	*ANXA8*	-2.2	-	-	-

ENSG00000	*ZFAND*	-2.3	-	+	-

ENSG00000	*KRT16*	-2.5	-	+	-

ENSG00000	*JMJD3*	-2.6	-	+	-

ENSG00000	*PLSCR*	-2.7	+	-	+

ENSG00000	*USP18*	-2.7	-	-	+

ENSG00000	*KRT17*	-2.7	-	-	-

ENSG00000127129	*EDN2*	-3.2	-	+	-

A search for SP1 Transcription Factor Binding Sites (TFBS) was performed on the promoter regions (-900/+100) of 157 TFAP2A-down-modulated genes [39], mapped in ENSEMBL, containing at least one or two best 20% TFAP2A binding sites (see Table 1) using the SP1 Positional Weight Matrix (PWM) provided by Jaspar. 57 genes containing SP1 TFBS were identified. The outcome obtained with the Oligo, oPOSSUM and MEME analyses are also indicated (see Table 4, 5, 6).

**Figure 6:**
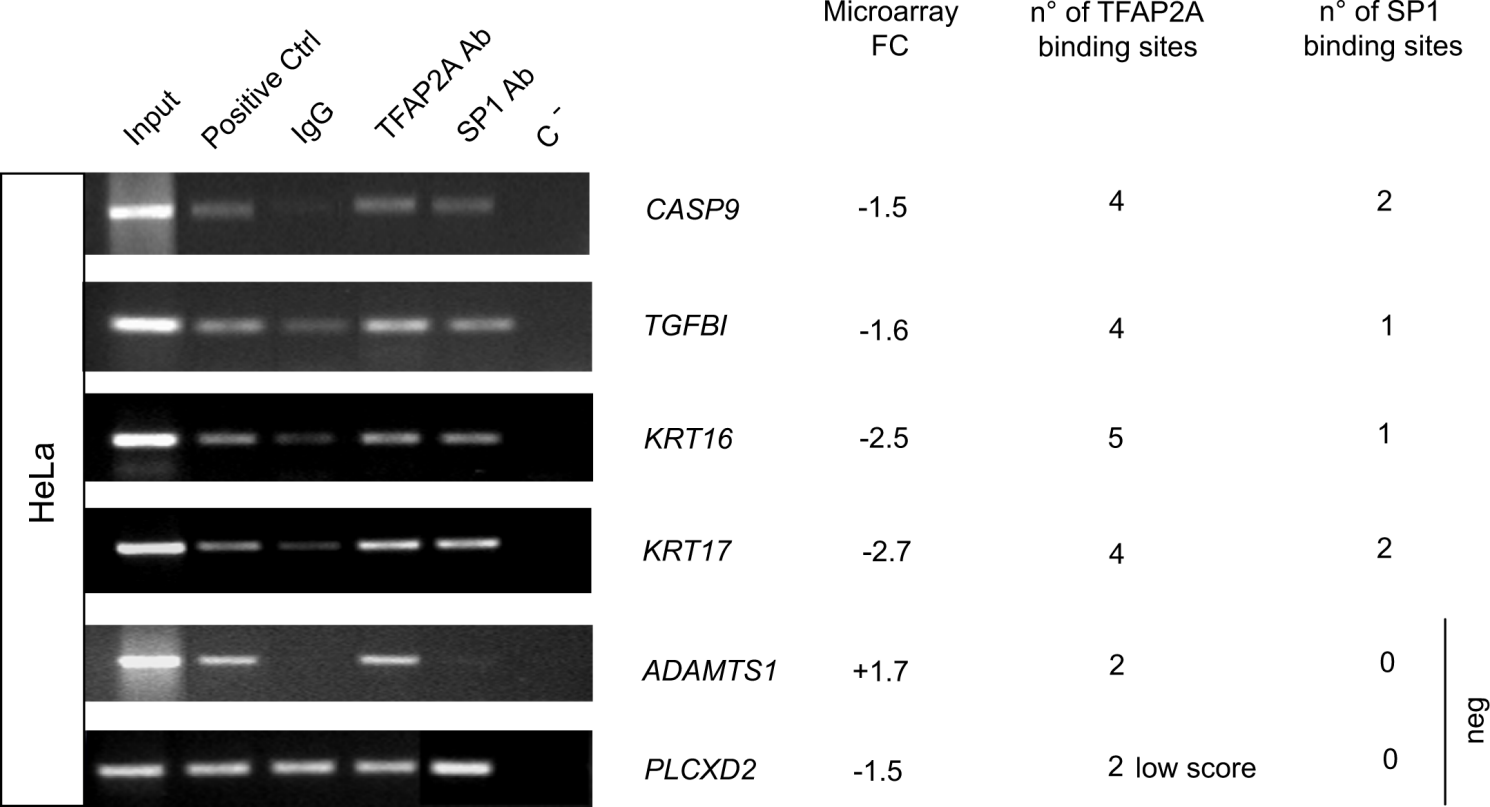
**In vivo Chomatin ImmunoPrecipitation (ChIP) analysis for putative SP1 binding sites on the core promoters of TFAP2A target genes**. Pre-cleared chromatin from HeLa cells was immunoprecipitated with either non-specific IgGs (IgG, negative control) or anti-RNA polymerase II or anti-acetylhistone H3 (positive controls: Positive Ctrl) or anti-SP1 (SP1) or anti-TFAP2A (TFAP2A) antibodies (Ab). Immunoprecipitated DNA or non-immunoprecipitated chromatin samples (Input) were amplified by PCR using primer pairs designed with the NCBI Primer Designing Tool (see Methods) across the TFAP2A or the SP1 putative binding sites. ***ADAMTS1***: ADAM metallopeptidase with thrombospondin type 1 motif, 1; ***CASP9***: caspase 9; ***KRT16***: keratin 16; ***KRT17***: keratin 17; ***PLCXD2***: pleckstrin homology-like domain family B member 2; ***TGFBI***: Transforming Growth Factor Beta-Induced. The number (n°) of computationally predicted high score TFAP2A or SP1 binding sites are indicated. C-, PCR negative control; FC, fold changes obtained from the microarray analysis [39]. Three independent experiments were performed and a representative one is shown here.

Potential SP1 binding was tested for 4 candidate target genes containing one or two best 20% SP1 and TFAP2A binding sites by Chromatin Immuno Precipitation (ChIP) assay, as indicated by Jaspar PWMs. See Figure 6. The 4 candidate genes were *CASP9 *(caspase 9); *KRT16 *(keratin 16); *KRT17 *(keratin 17) and *TGFBI *(Transforming Growth Factor B-Induced). ChIP analysis was performed on HeLa cells, using specific anti-SP1 and TFAP2A antibody. As shown in Figure 6, enrichment for SP1 together with TFAP2A was found on the promoter of each gene compared with negative controls, suggesting a functional role for both *cis*-elements. Negative controls for ChIP analysis were performed using genes where no high score binding sites for either SP1 or TFAP2A were identified such as *ADAMTS1 *(ADAM metallopeptidase with thrombospondin type 1 motif, 1) and *PLCXD2 *(pleckstrin homology-like domain family B member 2). Enrichment for TFAP2A but not for SP1 was found for *ADAMTS1 *while no enrichment for both TFs was observed for *PLCXD2*.

## Discussion

The results presented in this work show how powerful an *in silico *approach can be for the identification of functional Transcription Factor Binding Sites (TFBSs), in particular when computational investigations are associated with microarray analysis. In fact, while microarray analysis is, by definition, not able to discriminate between direct or indirect Transcription Factor (TF) gene expression modulation [45], the positioning of TFBSs on differentially expressed genes allows the identification of genes directly regulated by the TF of interest. By analyzing gene expression profiles on HeLa cells either silenced for TFAP2A by RNA interference or not [39] we were previously able to identify a subset of new TFAP2A regulated genes on which it was possible to position high score TFAP2A binding sites and find an enrichment of sites compared with genome-wide [5,6]. The strength of our computational approach was consolidated by experimental validations revealing TFAP2A binding to the high score TFAP2A sites identified but not to portions of DNA without TFAP2A binding sequences or containing only low score sites.

A network analysis performed with Ingenuity Pathway Analysis Systems for the TFAP2A-modulated genes containing at least two high score TFAP2A binding sites revealed "Cellular Movement" and "Cellular Development" as the main networks confirming results previously obtained in our and other laboratories. In fact, many reports demonstrated that TFAP2A plays a major role in development [12] and we recently showed a function for TFAP2A in cell migration and/or invasion for tumor cells [39] and neurons [46]. Genes involved in both biological processes were previously identified however several new ones have been discovered here. Among the already known genes, *PPARG*, *MAPK1*, and *VEGF *[47] are present in the networks confirming the validity of our analysis.

The "Cellular Movement" network includes genes with specific biological functions and some examples are listed here. *SLIT2 *(slit homolog 2 -Drosophila) is involved in induction of negative chemotaxis in neuronal cells, glial cell migration, motor axon guidance and nervous system development [48]. *PDGFs *(platelet-derived growth factors) are known to regulate cell proliferation as well as migration for mesenchymal or endothelial cells [49]. Interestingly, in vascular cells *PDGFBB *is known to up-regulate *DCBLD2/ESDN *(discoidin, CUB and LCCL domain containing 2/Endothelial and Smooth muscle cell Derived Neuropin-like molecule), the most TFAP2A-modulated gene in our microarray analysis. *PDGFBB *is also known to be related to another TFAP2A-modulated gene, *ACTA2 *(actin, alpha 2, smooth muscle, aorta), which codes for a protein belonging to the actin family that plays a role in cell motility, structure and integrity and regulates blood pressure via vascular and smooth muscle contraction [50]. *RAC1 *and *RAC2 *(ras-related C3 botulinum toxin substrate 1 and 2) are small GTPases belonging to the RAS superfamily and regulate a variety of cellular events, including growth control, cytoskeletal reorganization and protein kinase activation [51]. *PDGF *and *RAC1 *are connected with each other since it was demonstrated that all *RAC1*-related GTPases expressed in mouse primary fibroblasts, Cdc42, Rac1, and RhoG, are required for efficient migration following PDGF stimulation [52].

Some of the genes included in Cellular Development network are: *PPARs *(peroxisome proliferator-activated receptors) form heterodimers with retinoid X receptors (RXRs) and regulate the transcription of various genes. Three subtypes of *PPARs *are known: *PPARA*, *PPARD *and *PPARG*. The last one regulates adipocyte differentiation and is involved in the pathology of numerous diseases including obesity, diabetes, atherosclerosis and cancer [53]. *MAPK1 *(mitogen-activated protein kinase 1) is a member of the MAP kinase family and it is involved in cellular proliferation, differentiation, transcription regulation and development [54]. *CXCL1 *(chemokine, C-X-C motif, ligand 1 - melanoma growth stimulating activity, alpha) belongs to the Chemokine family, a group of small, structurally related molecules that regulate cell trafficking of various types of leukocytes via the interaction with a subset of 7-transmembrane, G protein-coupled receptors [55]. In addition, *CXCL1 *is known to play a major role in inflammation, angiogenesis, tumorigenesis and wound healing [56]. *ADAMTS1 *(a disintegrin-like and metalloprotease with thrombospondin type 1 motif, 1) gene encodes for a member of the ADAMTS protein family that has anti-angiogenic activity and the expression of this gene may be associated with various inflammatory processes as well as development of cancer and cachexia [57]. The protein encoded by the *AREG *(amphiregulin) gene is a member of the epidermal growth factor (EGF) family involved in cell growth stimulation of astrocytes, Schwann cells, fibroblasts and epithelial cells by interacting with EGF receptor [58]. *IL11 *(interleukin 11) encodes for a cytokine which stimulates the T-cell-dependent development of immunoglobulin-producing B cells and potentiates proliferation of hematopoietic stem cells and megakaryocyte progenitors [59].

Since it is well known that TFAP2A cooperates with other transcription factors (TFs) to regulate transcription, three different methods, over-represented oligonucleotides, oPOSSUM and MEME, were used to identify TF which could possibly work in cooperation with TFAP2A to regulate the 264 genes containing the best score TFAP2A binding sites. Remarkably a common enrichment for SP1 binding sites was found in genes containing at least one or two high score TFAP2A binding sites and transcriptionally activated by TFAP2A but not in the repressed ones. SP1 is known to cooperate with TFAP2A in transcription [60,61], however here we underline the importance of SP1 specifically in TFAP2A gene activation, but not in transcriptional repression and localize SP1 binding sites to DNA nucleotide sequences distinct from TFAP2A binding sites, although, from our experiments, we cannot exclude the possible physical interaction between TFAP2A and SP1.

Among the ChIP validated genes, the gene encoding for Discoidin, CUB and LCCL domain containing 2/Endothelial and Smooth muscle Derived Neuropilin like (*DCBLD2/ESDN*) resulted the most modulated in our microarray analysis with a (FC + 5.7). This is one of the reasons why we decided to investigate its TFAP2A-dependent transcription in detail together with its interesting functions. Its protein structure resembles that of neuropilins, transmembrane proteins which are promiscuous for ligands and co-receptors. *DCBLD2/ESDN *is ubiquitously expressed but linked to metastasis formation since it has been cloned and found to be significantly up-regulated from highly metastatic lung cancer cells [62]. Various functional studies link DCBLD2/ESDN to tumor progression but a specific role in tumor promotion or repression has not been defined yet [63,64]. *DCBLD2/ESDN *expression was analyzed in our (unpublished data) and other laboratories [63,65] in melanoma and breast cell lines and found to be expressed only in highly metastatic cells but not in their related poorly malignant variants suggesting a positive role for DCBLD2/ESDN in tumor progression. *DCBLD2/ESDN *was also shown to be part of an invasive breast cancer gene signature [66]. However in HeLa cells, used for our microarray analysis, we demonstrated that TFAP2A regulates tumor cell motility and invasion, at least partially, via *DCBLD2/ESDN *in a negative manner [39]. Here TFAP2A down-modulation prompted *DCBLD2/ESDN *up-regulation, suggesting a possible direct repressive effect of *DCBLD2/ESDN *transcription by TFAP2A. In our present investigation, overexpression of TFAP2A in cells expressing low or high levels of TFAP2A, respectively MDA-MD-231 and HeLa cells, led to decreased *DCBLD2/ESDN *promoter activity although in MDA-MB-231 cells *DCBLD2/ESDN *promoter activity was higher in comparison with HeLa cells. Accordingly TFAP2A silencing induced higher *DCBLD2/ESDN *promoter activity. Importantly, the negative effect of TFAP2A on *DCBLD2/ESDN *promoter was dose-dependent since when MDA-MB-231 cells were transfected with increasing levels of the TFAP2A-expression vector, a proportional down-regulation of transcription was observed. All together, these findings strongly suggest a direct repressive activity of TFAP2A on *DCBLD2/ESDN *promoter and are in agreement with our microarray results [39]. For these reasons we made the hypothesis that if TFAP2A represses *DCBLD2/ESDN *transcription, inverse expression profiles should exist for the two genes and therefore we used an on-line expression atlas for RNA expressions in tumors http://biogps.gnf.org. In some case high *DCBLD2/ESDN *expression coincided with very low levels of *TFAP2A *in tumor cells, while in other cases *DCBLD2/ESDN *high or low expression co-existed with high *TFAP2A *expression. In addition, for many tumors both genes showed comparable *TFAP2A *low or medium RNA levels. Since it is known that TFAP2 activity can be modulated by a wide range of interacting proteins [12], it is conceivable that differential expression and functional roles of TFAP2A co-factors may account for distinct effects on *DCBLD2/ESDN *gene transcription. Moreover, the presence of other TFAP2 isoforms and the relative ratios with one another may be crucial here. On the other hand, in many cases, both *TFAP2A *and *DCBLD2/ESDN *genes might not be expressed. Finally, it is important to keep in mind that RNA levels do not always correspond to actual protein levels or activity. For instance, TFAP2 proteins are known to be modified post-translationally by phosphorylation, sumoylation and redox status, which may affect their activity and cellular localization [12].

Three high and several low score TFAP2A binding sites were identified in the promoter region of the *DCBLD2/ESDN *gene by computational analysis however we only investigated the functional role and contribution of the main TFAP2A binding sequences. By doing so we observed that each TFAP2A site was essential for repression of *DCBLD2/ESDN *transcription, in fact the inactivation of one or two or three site/s equally affected promoter activity in luciferase assays. These experimental validations, confirm once again, that our computational analyses represent a powerful tool for the identification of TF regulatory targets by predicting precisely their *cis*-elements [19]. To better understand the repressive effect of TFAP2A on *DCBLD2/ESDN *transcription, the interaction of TFAP2A with other co-factors will be studied in the future. A better comprehension of the TFAP2A-driven regulation of the *DCBLD2/ESDN *gene should provide novel and useful insights on mechanisms of tumor progression and metastasis formation.

## Conclusions

Our study was essential for: 1) identifying functional TFAP2A binding sites in novel TFAP2A-regulated genes; 2) defining "Cellular Movement" and "Cellular Development" as the main networks in which the TFAP2A target genes are involved; 3) associate SP1 to TFAP2A gene transcription activation but not repression; 4) dissecting the TFAP2A-driven regulation of *DCBLD2/ESDN*, an important player of tumor progression.

## Methods

### Definition of promoter sequences and TFAP2A binding site identification genome-wide

Whole-genome human protein-coding gene sequences and annotations were downloaded from the ENSEMBL database, version 46, [67]. Only the longest transcript was considered for each gene and the promoter region defined as 900 bps upstream and 100 bps downstream of the Transcriptional Start Site (TSS), +900/-100 [68]. Each promoter sequence was analyzed using the canonical TFAP2A Position Weight Matrix (PWM) MA0003 reported in Jaspar database http://jaspar.genereg.net/ which consists of a 9 nucleotide GC-rich sequence. Affinity scores were assigned to each TFAP2A binding site using a standard log-likelihood ratio (LLR) scoring function with intergenic background frequencies. All sites with score exceeding 66% of the maximum possible score for the PWM were initially selected, then ranked by score. We considered the top ranking sites (the thresholds used were 10%, 20% and 30%) to identify genes carrying at least two high score sites in their regulatory region. The software described in [69] was used to rank the sites.

### Identification of TFAP2A potential co-factors in the (-900/+100) promoter regions using three different approaches

#### 1) Over-represented and Conserved Oligonucleotides (Table 4)

We first classified human and mouse genes in two categories (CG-rich and CG-poor) by analyzing the CG content of their promoters using the median CG content of the whole dataset as threshold. The two categories of genes were then independently searched for over-represented 5 to 9 bps-long oligonucleotides (oligos) where the over-representation was assessed using a binomial model [70] and the overall frequency *f(w) *of each oligo *w *was computed as:

where *N(w) *is the number of times that *w *occurs in the entire collection of sequences and . Instead *n*_*g*_*(w) *is the number of occurrences of *w *in the promoter region of each gene *g*. The statistical significance of over-representation was determined using the binomial P-value:

where

is the total number of oligos of the same length as *w *that can be found in the promoter region of *g. *Self-overlapping matches of the same oligo were discarded and motifs were counted on both DNA strands. For each oligo *w *we defined the set *S(w) *of the genes whose promoter shows overrepresentation of *w *(*P*_*g*_(*w*) < 0.01)

An oligo (*w*) was defined "conserved over-represented" if the sets of genes *S*_*human*_*(w) *and *S*_*mouse*_*(w) *contained a significantly larger number of orthologous genes than expected by chance. Pairs of human-mouse orthologous genes were obtained from ENSEMBL. In order to obtain one-to-one orthology relationships, only orthologous genes defined as Unique Blast Reciprocal Hit were considered. The significance of the overlap between *S*_*human*_*(w) *and *S*_*mouse*_*(w) *was determined with the exact Fisher test, and multiple testing taken into account by computing the False Discovery Rate (FDR) with the method of Benjamini and Yekutiely [71]. For further analysis we retained the oligos with FDR < 0.1. Additional details on this procedure can be found in [41,72]. In order to identify possible TFAP2A co-factors a direct comparison of the over-represented oligo sequences with the known consensus sequences for vertebrate Transciption Factors (TFs) [42] was performed and the association between motifs and TFs was accepted only if the over-represented oligo fully overlapped (according to the IUPAC alphabet).

#### 2) oPOSSUM (Table 5)

The oPOSSUM program [43] was used to identify Transcription Factor Binding Sites (TFBS) recognized by potential TFAP2A co-factors considering 60% sequence conservation between human and mouse as a minimum requirement. With this approach the regulatory region explored coincided with the smallest cut off we could choose (-2000/0) even if it included additional upstream bps compared with the over-represented oligo (see above) and MEME analyses (see below). The other parameters were left unchanged. A Fisher's exact test with a p_v _< 0.05 was performed here to identify the highly enriched TFBS [43].

#### 3) MEME (Table 6)

The MEME program [44] was used to identify TFAP2A potential co-factors considering 20 bps as the maximum length of any motif (to fit the standard size of a typical TFBS) and searching for motifs in both DNA strands. To assess whether the motifs obtained by MEME may be associated to any known TFBSs, each motif was associated to a putative TF based on [42].

### Localization of SP1 TFBS in the (-900/+100) promoter regions

Using the three approaches mentioned above it was possible to identify an enrichment for SP1 TFBS in the promoter regions of 157 TFAP2A-down-modulated genes mapped in ENSEMBL containing at least two high-score TFAP2A binding sites (see Table 1). In order to position the SP1 TFBS an additional analysis using the JASPAR PWM for SP1 (MA0079) was performed on the (-900/+100) promoter regions of this group of down-regulated genes, as described for TFAP2 (see above).

### Cell lines, Antibodies and DNA constructs

The following human cell lines were used; their origins and general properties, as illustrated by American Type Culture Collection (ATCC, Manassas, VA, USA), are as follows: HeLa: cervix adenocarcinoma (AC), HPV-18 positive; MDA-MB-231: breast AC, pleural effusion; HepG2 (Human Caucasian hepatocyte carcinoma). Each cell line was grown as suggested by ATCC. Primary antibodies used were anti-: TFAP2A mAb 3B5 or TFAP2A pAb C-18 or GAPDH pAb V-18 (Santa Cruz Biotechnology, Santa Cruz, CA) or SP1 pAb (Active Motif, Carlsbad, CA) or acetylated-H3 histone pAb (Upstate Biotechnology, Lake Placid, NY, U.S.A.). Secondary antibodies used were: goat anti-mouse or anti-rabbit IgG HRP-conjugated, donkey anti-goat IgG HRP-conjugated (Santa Cruz Biotechnology, Santa Cruz, CA); pSP(RSV)TFAP2A and pSP(RSV)-empty expression vectors, a gift from Dr. H. Hurst [73,74] were respectively used to overexpress human TFAP2A in cells and as empty vector control. TFAP2AshRNA2 [39] and pSUPER.retro.puro vector (OligoEngine, Seattle, WA, USA) were respectively used to down-modulate human TFAP2A in cells and as empty vector control.

### Molecular cloning of the human Endothelial and Smooth muscle Derived Neuropilin like *(DCBLD2/ESDN/CLCP1) *promoter

The upstream regulatory region of the *DCBLD2/ESDN/CLCP1 *(discoidin, CUB and LCCL domain containing 2/Endothelial and Smooth muscle cell Derived Neuropin-like molecule/CUB, LCCL-homology, coagulation factor V/VIII homology domains protein) gene was identified using the National Center for Biotechnology Information (NCBI) gene bank database (accession number NM_080927). A BAC genomic clone RZPDB737B122156D was purchased from imaGenes (imaGenes GmbH, Berlin, Germany) and a 2274 bps fragment encompassing the putative *DCBLD2/ESDN *promoter region was amplified by PCR, using Takara (Bio Inc., Shiga, Japan) reagents. A final 50 μL volume contained: 2.5 U Takara LA Taq enzyme, 1 × PCR Buffer, 400 μM dNTPs, 2.5 mM MgCl_2_, 1 μM forward and reverse primers and 0.1 μg of DNA. The following primers containing KpnI and BglII restriction sites at the 5' ends were used: FW: 5'-GGGGTACCCCCTGGCTGATTGGGGTTTTTA-3'; RV: 5'-GAAGATCTTCGCGGAGCTAAGGAACGTG-3'. A negative control without plasmid was always added. 3'-overhang As were added by a post-incubation with Taq polymerase (Invitrogen Life Technologies, Carlsband, CA) then the fragment was cloned into the pCR^®^2.1-TOPO vector using the TOPO TA cloning system (Invitrogen Life Technologies, Carlsband, CA). After sequencing, the fragment was excised by KpnI and BglII digestion and subcloned into pGL3-Basic Luciferase reporter vector (Promega, Madison, WI) giving rise to the pGL3-ESDN-WT reporter construct (-2185; +92). A 5'deletion construct was generated by digesting pGL3-ESDN-WT vector with KpnI-BalI restriction enzymes, followed by Klenow end-filling reactions (where necessary) and self-recircularization by Invitrogen ligase and named: pGL-ESDN-DEL3 (-950; +92). QuickChange™ Site-Directed Mutagenesis Kit (Stratagene, La Jolla, CA) was used to generate 7 bp-deletions in the central portion of every single TFAP2A binding site in single or multiple combinations and pGL3-ESDN-MUT1, pGL3-ESDN-MUT2, pGL3-ESDN-MUT3, pGL3-ESDN-MUT1,2, pGL3-ESDN-MUT1,3, pGL3-ESDN-MUT2,3, pGL3-ESDN-MUT1,2,3 reporter vectors were obtained. Mutagenic primer sequences are listed in Additional file 2.

### Chromatin ImmunoPrecipitation (ChIP) assays

ChIP was performed using the Magna ChIP™ G kit (Millipore, Billerica, MA) reagents and protocols. Briefly, chromatin was prepared from HeLa or HepG2 cells at ~80-90%. Cells were crosslinked with 1% formaldehyde (Sigma Aldrich, St Louis, MO) for 10' at 37°C. Chromatin shearing was obtained by digesting the DNA with an enzymatic shearing cocktail (200 U/ml) for 10' at 37°C. Chromatin was pre-cleared with Protein G beads, to reduce non-specific background. Pre-cleared chromatin was immunoprecipitated overnight using 2 μg of specific antibodies. In addition, chromatin aliquots were precipitated with either non-specific IgGs or with anti-RNA polymerase II or anti-acetyl-histone H3 antibody, used respectively as negative and positive controls. Immunoprecipitated chromatin was collected by adding protein G beads to the tubes and the beads were washed with ChIP-IT™ Washing Buffers supplemented with protease inhibitors. Immunoprecipitated DNA was collected and after reversing the cross-links, DNA was purified by using the QIAquick^® ^PCR Purification Kit mini spin-columns (QIAGEN, Stanford CA), according to manufacturer's instructions. The eluted immunoprecipitated DNA was analyzed by PCR, together with a non-immunoprecipitated chromatin sample (input). Polymerase chain reaction (PCR) was performed using Taq DNA Polymerase (Invitrogen Life Technologies, Carlsband, CA) using 1 × PCR Buffer without MgCl_2_, 0.2 mM dNTPs, 1.5 mM MgCl_2_, 0.5 μM forward and reverse primers, 0.625 U Taq DNA Polymerase and 10 μL of precipitated DNA. The annealing temperature and the number of cycles were specific for each primer pair. The different primer pairs were designed using the NCBI Primer designing tool and primer sequences as well as PCR experimental conditions are added in Additional file 2. To verify the quality of our ChIP reactions, primers to the *GAPDH *promoter were used as positive controls.

### Transient transfections and luciferase assays

Twenty-four hours before transfection, HeLa or MDA-MB-231 cells were seeded in 24-well plates at 8 × 10^4 ^cells per well. Cells were transfected using Lipofectamine 2000 (Invitrogen Life Technologies, Carlsband, CA) and 700 ng of either pGL3-Basic (control) or the various pGL3- *DCBLD2/ESDN *promoter fragments generated and mentioned above in presence of 20 ng of pRLTK (Promega, Madison, WI) to normalize for transfection efficiency following the manufacturer's instructions. In co-transfection experiments, 250 ng of pSP(RSV)TFAP2A or pSP(RSV)NN or TFAP2AshRNA2 or pSUPER.retro.puro vectors were used. Forty-eight hours after transfection cell extracts were prepared by adding 100 μl of 1 × Passive Reporter Lysis Buffer (Promega, Madison, WI). The luciferase activities were measured using the Dual Luciferase Assay System (Promega, Madison, WI) according to the manufacturer's instructions. Each transfection was performed in triplicate and repeated three times. For the statistical analysis a student's *t *test was performed: * p_v _< 0.05; ** p_v _< 0.01; *** p_v _< 0.001.

### Protein preparation and immunoblotting

Total protein extracts were prepared using a boiling Laemli Buffer containing 0.125 M Tris/HCl, pH 6.8 and 2.5% SDS. 25 μg of proteins were separated by 12% SDS-PAGE and electroblotted onto PVDF membranes (Bio-Rad, Hercules, CA). Membranes were blocked in 5% non-fat milk-TBS-Tween buffer (137 mM NaCl, 20 mM Tris/HCl, pH 7.6, 0.1% Tween-20), overnight at 4°C, then incubated with appropriate antibodies for 1 hour at room temperature and visualized by enhanced chemiluminescence (ECL^®^, Amersham Biosciences, Pisactaway, NJ).

### Ingenuity Pathway Analysis Systems

The Ingenuity Pathways Knowledge Base http://www.ingenuity.com is currently the world's largest database of knowledge on biological networks, with annotations organized by experts. We exploited this database to define the presence of functional associations within the genes detected by microarray analysis, to identify enriched ontological gene classes and to draw simplified network connections among genes. Each network was ranked based on "scores" which consider how relevant the networks are to the genes in our input dataset. Each score is based on a p-value calculation, which takes in account the probability that the genes present in a network are found in it just by chance. Mathematically, the score is simply the negative exponent of the right-tailed Fisher's exact test result.

## Abbreviations

TFs: transcription factors; PWM: Positional Weight Matrix; ChIP: Chromatin ImmunoPrecipitation; TFBSs: TF Binding Sites; PFM: Positional Frequency Matrix; TSS: Transcriptional Start Site; GO: Gene Ontology; FC: Fold Change.

## Authors' contributions

FO participated in the design and coordination of the study, performed the ChIP-analyses and drafted the manuscript. DC participated in the design and coordination of the study and performed bioinformatics analyses. BU performed the ChIP and ESDN-promoter analyses and participated in writing the manuscript. PP supervised the bioinformatics analyses. MC supervised the bioinformatics analyses, interpreted the bioinformatics results and contributed to the organization of the manuscript. DT participated in the design and coordination of the study, interpreted the results, drafted the manuscript and supervised the revision. All authors read and approved the final manuscript.

## Supplementary Material

Additional file 1 **Microarray analysis (Whole Human Genome Agilent 44 K) was performed on HeLa cells transiently transfected with either generic non-silencing (NS) or specific TFAP2A (oligo TFAP2A) siRNA oligos**. The accession numbers of 494 modulated gene [39] were unambiguously converted to ENSEMBL IDs (version 46) and used for our analysis as listed here. Click here for file

Additional file 2 **List of primers and PCR conditions used for cloning, mutagenesis and ChIP assays**. Click here for file
