# Supplementary material for: Identification of functional TFAP2A and SP1 binding sites in new TFAP2A-modulated genes
Source: BMC Genomics. 2010 Jun 3;11:355. doi: 10.1186/1471-2164-11-355 (PMC2890567; doi:10.1186/1471-2164-11-355)
Supplement: Additional file 2 — List of primers and PCR conditions used for cloning, mutagenesis and ChIP assays. [file 1471-2164-11-355-S2.DOC]

Additional file 2

| **Primer** | **Sequence** | **T annealing** | **Cycles** |
| --- | --- | --- | --- |
| **PLCXD2 FW** | CCCACAGTCCTGCCATCTGA | 60° C | 36 |
| **PLCXD2 RV** | CCCCAAGCATGAAGGTGAGG |
| **IFI44 FW** | GCTATTGTTTTTAATAGGGCCAGCA | 60° C | 36 |
| **IFI44 RV** | GGCAGAATTCAATCCGCACA |
| **SLIT2 FW** | GGGGACGGAATTCAAAGCCT | 60° C | 36 |
| **SLIT2 RV** | AGTAGTGTGTGCGCGCGTGT |
| **ADAMTS1 FW** | TCCGCCCCTAGATTGACGAG | 60° C | 36 |
| **ADAMTS1 RV** | TTTGCAATAGCCCCTGGCTC |
| **CXCL1 FW** | TTACTCTGAAGGGCGAGGCG | 60° C | 36 |
| **CXCL1 RV** | GAAGAGCTGGCGAGGAGGTG |
| **CASP9 FW** | GTGACGCAAGAGCGAATCCTT | 60° C | 36 |
| **CASP9 RV** | CAGGGCCAAGCCTCCCAT |
| **ESDN/DCBLD2 FW** | CGGAGAACTAGCGGGTCGAG | 62° C | 32 |
| **ESDN/DCBLD2 RV** | GGAGGGAAGGAAGCGGAGTC |
| **EREG FW** | GTCCACGCTCCCCACCTC | 60° C | 36 |
| **EREG RV** | CGTTGGCTGTGTCCCTCTGA |
| **CD59 FW** | CCCAAGCCTCGCAGTAGGAA | 55° C | 36 |
| **CD59 RV** | CCAGGACCCAATGAGCACCT |
| **GLO1 FW** | GGGACCCAATCAGCAGAGAGTC | 60° C | 36 |
| **GLO1 RV** | CTGTGCCCACCTTGGTACTGG |
| **PPARG FW** | GCAGCACCACCGATCAGAAG | 59° C | 36 |
| **PPARG RV** | GCCCAGAGAGGGTCCCATTT |
| **KRT16 FW** | TGGGGCAGGGAGTTCTTCTG | 59° C | 36 |
| **KRT16 RV** | TGCGAGTTCGGTGGTGACTC |
| **KRT17 FW** | TCCGAGGCTTGATGGCATTT | 60° C | 36 |
| **KRT17 RV** | CCTCAACCCTGTCTGGTGGG |
| **TGFBI FW** | AGCCACAGGAGGCCTAAGGG | 60° C | 36 |
| **TGFBI RV** | GTGCCCGTGCTCAGTTTTCC |
| **FASTK FW** | GCACTGGGAGTGGGCTCCT | 60° C | 36 |
| **FASTK RV** | CCCGCACACACTCAGACACA |
| **ESDN/DCBLD2 cloning FW** | GGGGTACCCCCTGGCTGATTGGGGTTTTTA | touchdown  72°-65° C | 31 |
| **ESDN/DCBLD2 cloning RV** | GAAGATCTTCGCGGAGCTAAGGAACGTG |
| **ESDN-MUT1 FW** | GCGCCCGGGGGGGCCGC | 55°C | 18 |
| **ESDN-MUT1 RV** | CGCGGCCCCCCCGGGCGC |
| **ESDN-MUT2 FW** | GCCGGGCGGGGGGATCCGAAGCTG | 55°C | 18 |
| **ESDN-MUT2 RV** | CAGCTTCGGATCCCCCCGCCCGGC |
| **ESDN-MUT3 FW** | CGAGGGAGCTGGCCTGCCTGCCAG | 55°C | 18 |
| **ESDN-MUT3 RV** | CTGGCAGGCAGGCCAGCTCCCTCG |
| **ESDN-MUT1,2 FW** | GGCGCCCGGGGGGGGATCCG | 55°C | 18 |
| **ESDN-MUT1,2 RV** | CGGATCCCCCCCCGGGCGCC |
